# Clinical prediction model for prognosis in kidney transplant recipients (KIDMO): study protocol

**DOI:** 10.1186/s41512-022-00139-5

**Published:** 2023-03-07

**Authors:** Simon Schwab, Daniel Sidler, Fadi Haidar, Christian Kuhn, Stefan Schaub, Michael Koller, Katell Mellac, Ueli Stürzinger, Bruno Tischhauser, Isabelle Binet, Déla Golshayan, Thomas Müller, Andreas Elmer, Nicola Franscini, Nathalie Krügel, Thomas Fehr, Franz Immer, the Swisstransplant Kidney Working Group (STAN), Patrizia Amico, Patrick Folie, Monique Gannagé, Maurice Matter, Jakob Nilsson, Andrea Peloso, Olivier de Rougemont, Aurelia Schnyder, Giuseppina Spartà, Federico Storni, Jean Villard, Urs Wirth-müller, Thomas Wolff, the Swiss Transplant Cohort Study, John-David Aubert, Vanessa Banz, Sonja Beckmann, Guido Beldi, Christoph Berger, Ekaterine Berishvili, Annalisa Berzigotti, Pierre-Yves Bochud, Sanda Branca, Heiner Bucher, Emmanuelle Catana, Anne Cairoli, Yves Chalandon, Sabina De Geest, Sophie De Seigneux, Michael Dickenmann, Joëlle Lynn Dreifuss, Michel Duchosal, Sylvie Ferrari-Lacraz, Christian Garzoni, Nicolas Goossens, Jörg Halter, Dominik Heim, Christoph Hess, Sven Hillinger, Hans H Hirsch, Patricia Hirt, Linard Hoessly, Günther Hofbauer, Uyen Huynh-Do, Bettina Laesser, Frédéric Lamoth, Roger Lehmann, Alexander Leichtle, Oriol Manuel, Hans-Peter Marti, Michele Martinelli, Valérie McLin, Aurélia Merçay, Karin Mettler, Nicolas J Mueller, Ulrike Müller-Arndt, Beat Müllhaupt, Mirjam Nägeli, Graziano Oldani, Manuel Pascual, Jakob Passweg, Rosemarie Pazeller, Klara Posfay-Barbe, Juliane Rick, Anne Rosselet, Simona Rossi, Silvia Rothlin, Frank Ruschitzka, Thomas Schachtner, Alexandra Scherrer, Macé Schuurmans, Thierry Sengstag, Federico Simonetta, Susanne Stampf, Jürg Steiger, Guido Stirnimann, Christian Van Delden, Jean-Pierre Venetz, Julien Vionnet, Madeleine Wick, Markus Wilhelm, Patrick Yerly

**Affiliations:** 1 Swisstransplant, Bern, Switzerland; 2 Department of Nephrology and Hypertension, Inselspital, Bern University Hospital, Bern, Switzerland; 3 Department of Medicine, Division of Nephrology, University Hospital of Geneva, Geneva, Switzerland; 4 Nephrology and Transplantation Medicine, Kantonsspital St. Gallen, St. Gallen, Switzerland; 5 Clinic for Transplantation Immunology and Nephrology, University Hospital Basel, Basel, Switzerland; 6 STCS Patient Advisory Board, University Hospital Basel, Basel, Switzerland; 7 Transplantation Center, Lausanne University Hospital, Lausanne, Switzerland; 8 Department of Nephrology, University Hospital Zurich, Zurich, Switzerland; 9 Department of Internal Medicine, Cantonal Hospital Graubünden, Chur, Switzerland

**Keywords:** Prediction model, Prognostic model, Prognosis, Risk calculator, Risk score, Kidney transplantation, Graft survival, Quality of life, Patient-reported health status, Estimated glomerular filtration rate, eGFR

## Abstract

**Background:**

Many potential prognostic factors for predicting kidney transplantation outcomes have been identified. However, in Switzerland, no widely accepted prognostic model or risk score for transplantation outcomes is being routinely used in clinical practice yet. We aim to develop three prediction models for the prognosis of graft survival, quality of life, and graft function following transplantation in Switzerland.

**Methods:**

The clinical kidney prediction models (KIDMO) are developed with data from a national multi-center cohort study (Swiss Transplant Cohort Study; STCS) and the Swiss Organ Allocation System (SOAS). The primary outcome is the kidney graft survival (with death of recipient as competing risk); the secondary outcomes are the quality of life (patient-reported health status) at 12 months and estimated glomerular filtration rate (eGFR) slope. Organ donor, transplantation, and recipient-related clinical information will be used as predictors at the time of organ allocation. We will use a Fine & Gray subdistribution model and linear mixed-effects models for the primary and the two secondary outcomes, respectively. Model optimism, calibration, discrimination, and heterogeneity between transplant centres will be assessed using bootstrapping, internal-external cross-validation, and methods from meta-analysis.

**Discussion:**

Thorough evaluation of the existing risk scores for the kidney graft survival or patient-reported outcomes has been lacking in the Swiss transplant setting. In order to be useful in clinical practice, a prognostic score needs to be valid, reliable, clinically relevant, and preferably integrated into the decision-making process to improve long-term patient outcomes and support informed decisions for clinicians and their patients. The state-of-the-art methodology by taking into account competing risks and variable selection using expert knowledge is applied to data from a nationwide prospective multi-center cohort study. Ideally, healthcare providers together with patients can predetermine the risk they are willing to accept from a deceased-donor kidney, with graft survival, quality of life, and graft function estimates available for their consideration.

**Study registration:**

Open Science Framework ID: z6mvj

## Introduction

Kidney failure affects between 5 and 7 million people worldwide [1]. Kidney transplantation is considered as the best possible renal replacement therapy. In Switzerland, approximately 240 deceased-donor kidneys are transplanted each year, with approximately 1400 to 1500 patients on the waiting list [2]. Patients receiving a kidney transplant have overall lower mortality compared to patients on dialysis [3, 4].

Nevertheless, despite considerable improvements in the last decades, patients continue to experience late allograft failure. In Switzerland, kidney transplant recipients are enrolled in a nationwide prospective cohort (Swiss Transplant Cohort Study; STCS), with longitudinal follow-up of allograft and patient outcomes after transplantation [5, 6]. However, a validated prognostic model for the risk of allograft failure or patient-reported outcomes such as the quality of life is lacking in Switzerland. Accurately predicting individual patient outcomes would not only be relevant for clinical and therapeutic care, but also enable quality control and the optimization of organ transplantation programs. This can further advance the patients’ gained life-years.

According to a systematic review on risk prediction models for graft survival after kidney transplantation, the most common predicted outcome was graft survival, either with graft failure and death as event (composite endpoint) or with graft failure as event and censored for death [7]. However, with an aging population and an increase in elderly transplant recipients, death with a functioning graft can increasingly bias the results and should rather be considered as a competing risk [8, 9].

Most prediction models for outcomes in kidney transplantation have around 4 to 14 predictor variables [10–14]; often the models consider either donor-only or recipient-only characteristics, but combining both donor and recipient variables in the model may further improve the prognosis [9]. Among the most prominent risk scores is the KDPI (kidney donor profile index) released by the OPTN (US Organ Procurement & Transplantation Network) using 10 donor-only predictors [14, 15]. However, this score would prove suboptimal in clinical practice in Switzerland as some predictors (e.g., ethnicity, hypertension, diabetes, hepatitis C status) do not vary as much when compared to the USA population. Furthermore, the organ transport and related ischemia times are shorter in Switzerland and marginal donor kidneys (lower quality organs with still acceptable medical risks) are transplanted more often compared to the USA. Therefore, due to the numerous differences in donor organ quality, transplant setting, and recipient characteristics a risk score cannot be easily adapted in clinical practice.

Another disadvantage of existing prediction models is that they mainly consider hard endpoints such as patient survival and graft failure; however, these are late events and alternative endpoints are needed that can be assessed early in time. Patient-reported health-related quality of life may be at least as relevant [16, 17] and is closely associated with mortality and survival in end-stage renal disease [18, 19]. Furthermore, new surrogate endpoints for kidney graft survival have recently been suggested such as the slope of the estimated glomerular filtration rate (eGFR slope) for kidney disease progression [20–22].

In summary, a number of prognostic models have been proposed to predict kidney graft survival [9–14]. However, due to variability in donor mixes (donation after brain death [DBD] vs. donation after circulatory death [DCD]), transplant settings (e.g., ischemia times), patient population characteristics, timepoint of risk calculation, and substantial variability in the data available across different countries and the restriction to hard endpoints existing models cannot easily be adapted into clinical practice in Switzerland.

### Objective

The main objective of this study is to develop and validate three clinical kidney prediction models (KIDMO) for graft survival (primary outcome), quality of life, and eGFR slope (secondary outcomes) that included both donor and recipient characteristics as predictors. The study includes model development, internal-external cross-validation and is based on high-quality clinical data from a prospective national multi-centre cohort study and computable at the time of organ allocation. This novel prognostic tool could enable a reliable and valid prognosis at the time of organ allocation compared to existing clinical risk scores. Additionally, existing prognostic models can be assessed with respect to potential bias by means of model recalibration and model revision and subsequently be used as a benchmark for the novel prognostic tool.

## Research design and methods

The study protocol and related materials (expert survey and the a priori selected candidate predictors) are available on the project page [23] (osf.io/35apn) and have been registered on 1 September 2022 on the Open Science Framework (osf.io/z6mvj).

### Data source

In Switzerland, the STCS prospectively enrolls all solid organ transplant recipients since 2008 at six transplantation centres (Basel, Bern, Geneva, Lausanne, St. Gallen, and Zurich) [5, 6]. In order to successfully implement the prediction models, data from two data sources need to be combined:

1. SOAS database (Swiss Organ Allocation System) performs organ allocation according to the Swiss federal law and includes donor, transplant, and recipient characteristics (donor age, sex, cause of death, creatinine, ischemia time, donor-recipient immunological assessments, etc.)
2. STCS database (Swiss Transplant Cohort Study) longitudinally collects patients’ mid and long-term outcomes (status [death, alive, lost to follow-up], graft loss date, quality of life assessment, creatinine, etc.) at baseline, 6 months, and every 12 months after transplantation.

**Fig. 1:**
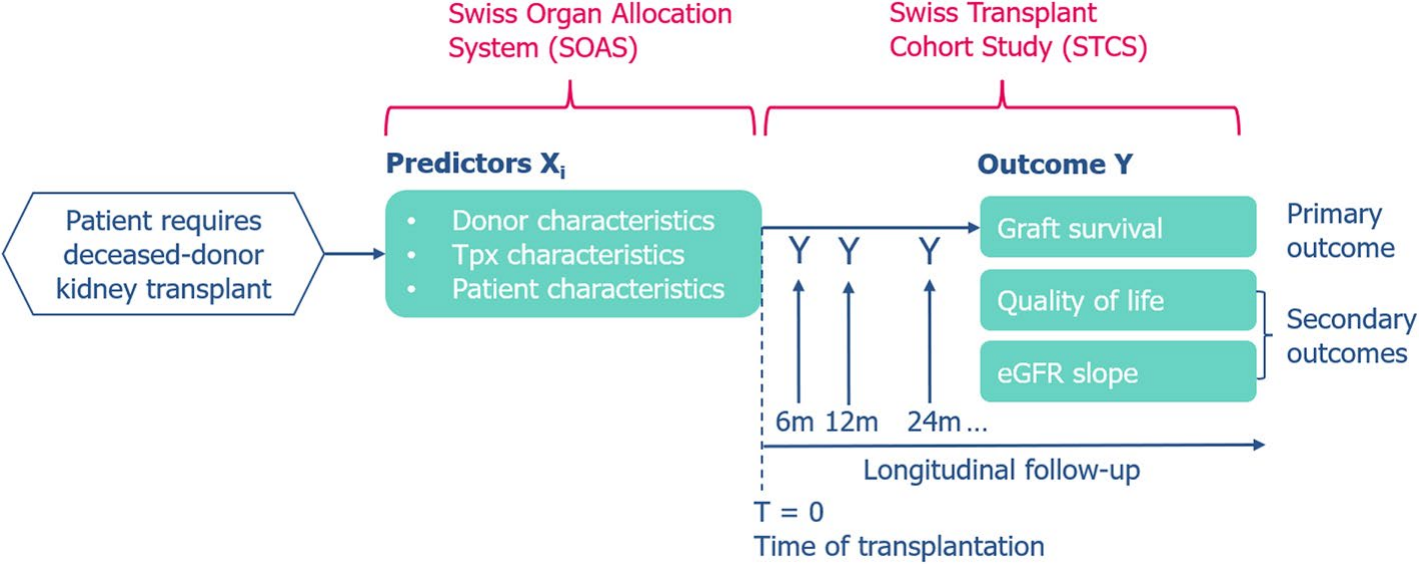
Schematic overview of the multivariable prediction models and the data sources involved. Three separate models will be developed to predict the three outcomes by a set of predictors. Predictors are based on SOAS data, while mid- and long-term outcomes were collected by a national multi-centre cohort study

SOAS data is provided by the Federal Office of Public Health, and STCS data by the STCS Data Centre after peer-review of the proposal and approval by the local ethics committee (KEK Bern). Donor-recipient data linkage is ensured via the unique recipient identifier (SOAS RS-number). A schematic overview of the prediction models and the data involved is shown in Fig. 1.

### Target population

We will include all kidney transplant recipients from 2008 onwards that were prospectively enrolled by the STCS and gave informed consent. Exclusion criteria is living-donor transplantation. Multi-organ transplantation, pediatric patients, and pre-emptive transplantations may be considered for exclusion if there were not enough samples and events in these subpopulations (see also “Sensitivity analyses”).

### Study outcomes

Our three outcomes of interest are defined as follows:

- Primary outcome: death-censored graft survival [24, 25] calculated from the date of transplantation to the date of irreversible graft failure by return to dialysis (or retransplantation). Death with a functioning graft is considered as a competing risk.
- Secondary outcomes: quality of life (self-reported overall health) at 12 months with the EQ-VAS [26–28] and kidney disease progression with eGFR slope [ml/min per 1.73m^2^/year] assessed using the first two eGFR follow-up measurements (6 and 12 months) calculated according to the Chronic Kidney Disease Epidemiology Collaboration (2021 CKD-EPI) [29] which is based on serum creatinine, age, and sex.

### Clinical predictor variables

We used expert knowledge to prespecify candidate predictors for graft survival (primary outcome); the same candidate predictors will also be used for the secondary outcomes. For this purpose, a kidney expert group was formed (4 transplant nephrologist from different Swiss transplant centres). Firstly, a comprehensive list of variables was created based on expert interviews and published prediction models. Secondly, a survey (osf.io/gc7jt) was performed (*N*=8, six transplant nephrologists and two kidney transplant recipients) to rate each variable on a 5-point Likert scale (not relevant, low relevance, neutral/not sure, relevant, very relevant). Altogether, 62 variables were assessed for relevance and a list was created with the variables that had the highest average score. Third, the list was presented to the expert group to determine the final candidate predictors (osf.io/f972e), taking into account the score from the survey, clinical considerations, data availability at time of allocation, overlap with other variables, and the number of coefficients to be estimated based on the sample size calculation (as described below). Fourth, the final candidate predictors were presented to the Swisstransplant kidney working group (STAN) that involves kidney experts from all the six transplant centres for final discussion. The final set of candidate predictors are shown in Table 2 and include 16 variables with 19 coefficients to be estimated.

### Sample size

Guidelines regarding the minimum required sample size for the development of a new multivariable prediction model have been proposed [30]. We used the accompanying R package “pmsampsize” [31]: the approach uses a set of criteria to minimize overfitting and ensure precise estimation of key parameters in the prediction model; a R notebook with code is available online (osf.io/35apn).

Based on information from the STCS data centre there were a total of *N*=2537 kidney transplantations between 2008 and 2020 (excluding all living-donor transplantations), see Table 1. Subtracting 20% of the data for cross-validation, adding 240 additional patients enrolled in 2021, and assuming 5% missing data, we arrived at an available sample size of *N*=2110 patients for model development for graft survival and eGFR slope, and *N*=1126 for quality-of-life self-assessments at 1-year follow-up (due to lower return rate). We then calculated the number of parameters that seem feasible to estimate in a multivariable model given the data.

**Table 1:** Number of kidney transplantations (deceased donors) per year and transplantation center

	2008	2009	2010	2011	2012	2013	2014	2015	2016	2017	2018	2019	2020	Sum	%
BE	17	35	28	25	18	20	25	26	19	25	42	36	29	345	14%
CHUV	17	24	26	19	28	17	33	38	33	35	34	34	32	370	15%
HUG	19	18	23	16	17	24	17	27	18	25	29	26	18	277	11%
KSSG	11	16	13	19	12	12	16	12	16	18	9	13	8	175	7%
USB	49	39	30	37	24	33	33	47	33	48	60	41	42	516	20%
USZ	54	56	57	68	61	63	64	73	66	81	70	69	72	854	34%
Sum	167	188	177	184	160	169	188	223	185	232	244	219	201	2537	100%

*BE* Inselspital, Bern University Hospital, *CHUV* Centre hospitalier universitaire vaudois (Lausanne), *HUG* Hôpitaux Universitaires de Genève, *KSSG* Kantonspital St. Gallen, *USB* University Hospital Basel, *USZ* University Hospital Zurich

For the primary outcome graft survival, we calculated a Cox-Snell adjusted *R*^*2*^ based on the information reported in a study with the same primary outcome and a similar population [13]. The study reported a C statistic of 0.78, 241 events, a sample size of *N*=2169 and 11 parameters. This resulted in a Cox-Snell *R*^*2*^ of 0.08 [32]. We used a graft failure rate of 0.017 and a median follow-up of 4.8 years; this information was derived from Table 2 in [6]. For a model with 19 parameters and a shrinkage factor of 0.9, the resulting minimum sample size was *N*=2042. The anticipated number of events were 167 which corresponded to 8.8 events per parameter.

**Table 2:** Clinical candidate predictors for model development with the number of coefficients to be estimated. The sum of the number of coefficients to be estimated was based on a sample size calculation for clinical prediction models

Nr	Variable type	Variable name	Level of measurement	Coefficients	Comments
1	Donor	Age	Continuous	3	Restricted cubic splines with 3 knots
2	Donor	Donor type (DCD, DBD)	Binary	1	Donation after circulatory death (DCD) or brain death (DBD)
3	Donor	History of diabetes	Binary	1	
4	Donor	History of hypertension	Binary	1	
5	Donor	eGFR on admission	Continuous	1	Estimated glomerular filtration rate
6	Donor	eGFR at allocation	Continuous	1	
7	Donor	Resuscitation	Binary	1	Donor was reanimated or not
8	Donor	Cause of death	Categorial	2	Using the 2 most common causes
9	Transplantation	Anti-HLA: DSA	Binary	1	Presence of donor-specific antibodies
10	Transplantation	HLA mismatches	Count	1	Human leukocyte antigen mismatches; number between 0 and 6
11	Transplantation	Retransplantation	Binary	1	Whether recipient was retransplanted
12	Recipient	Age	Continuous	1	
13	Recipient	Cardiovascular disease	Binary	1	
14	Recipient	History of diabetes	Binary	1	
15	Recipient	BMI	Continuous	1	Body mass index
16	Recipient	Pre-transplant dialysis	Continuous	1	Time on dialysis
			**Total:**	**19**	

We also performed sample size calculations for the secondary outcomes. For the quality of life (continuous), we anticipated an adjusted *R*^*2*^ of 0.15 as a lower bound for the model. This estimate is conservative as a study with the same outcome reported an *R*^*2*^ of 0.35, however, in a different population [33]. We know from a previous study that the EQ-VAS score has a mean (SD) of 62.8 (20.73) in the Swiss kidney transplant population [34]. A model with a shrinkage factor of 0.9, an anticipated *R*^*2*^ of 0.15, and 20 parameters required a minimum sample size of *N*=984; for 50 parameters and an *R*^*2*^ of 0.35 the sample size was *N*=996. For the eGFR slope, we anticipated an *R*^*2*^ of 0.15 and a mean (SD) from two published studies of 1.28 (2.5) and 1.8 (1.9), respectively [22, 35]. In both scenarios, a model with 40 parameters resulted in a minimum sample size of *N*=2090.

Therefore, the following number of parameters can be reliably estimated in a prognostic model:

- Graft survival: 19 parameters
- Health state (EQ-VAS): 20–50 parameters
- eGFR slope: 40 parameters

### Statistical analysis

Patients’ baseline characteristics will be reported using mean and standard deviation for continuous variables, median and interquartile range in case of non-normality (assessed with histograms), and absolute and relative frequencies for categorial variables. Missing data will be assessed and reported for each variable. As the fraction of missing data is expected to be below 5%, a complete case analysis will be carried out. All analyses will be performed with the R software for statistical computing version 4.2.2 [36].

### Prediction model development

For the primary outcome graft survival (time-to-event data), we will use a Fine & Gray model which is an extension to the Cox model to address competing risks using the functions coxph() and finegray() from the survival package [37, 38]. Our research question is focused on the direct assessment of the actual risk. Thus, a regression model that directly acts on cumulative incidence function (CIF) is to be preferred over cause-specific hazards in the context of prediction, the estimation of absolute risks, and clinical decision making [39–42]. For the secondary outcomes, quality of life and eGFR slope (continuous data), we will use two linear mixed models.

Dependencies in the data (kidney allografts from the same donor and retransplanted recipients) will be addressed as follows: as the function coxph() only supports a single cluster term, we will use exploratory analyses to determine which is more important of donor ID or recipient ID. A cluster term in the Fine & Gray model and a random intercept term in the mixed model will then account for dependencies in the data.

After we fitted a model using the a priori selected candidate predictors (Table 2), we perform model reduction with backward elimination using the Akaike information criterion (AIC) [43]. This step is repeatedly done using bootstrap resampling [44, 45], and candidate predictors are required to be retained in > 50% of the bootstrap samples.

Throughout model development, we will perform the following model diagnostics:

- Investigating potential nonlinear relationship between continuous variables and the outcome with restricted cubic splines
- Checking multicollinearity among predictors (using variance inflation factor; VIF)
- Checking proportional hazards assumption with Schoenfeld residuals
- Inspection of residuals, i.e., residuals vs. fitted values, comparing residual variance across study centres, and Q-Q plots

Coefficient estimates of the predictors and 95% CIs will be determined and discussed with the expert group for clinical interpretability.

### Model evaluation and internal-external cross-validation

For the primary outcome, model evaluation includes cumulative incidence curves for different risk groups based on the prognostic index, calibration plots, calibration intercept and slope, Brier score, and Harrell’s c-statistic [8, 46]. For the secondary outcomes, we assess the root-mean-square error (RMSE), the explained variation statistic (adjusted *R*^*2*^), and calibration plots.

For internal validation, we use Monte-Carlo bootstrapping [47]: Models will be developed on 200 bootstrap samples and tested on the same and on the original samples to assess optimism-corrected performance [44]. In a next step, we use internal-external cross-validation [45] with the original data from every transplant center being left out once for validation of the model that was based on data from the remaining centres. This will allow us to assess heterogeneity across transplant centres with random effects meta-analysis [48]. After validation, the final model will be fitted on all the available data and will also include a fixed effect term for the transplant centers.

### Sensitivity analyses

In a sensitivity analysis, we will define an eGFR < 15ml/min/1.73m^2^ as kidney failure in line with the KDIGO 2012 clinical practice guidelines and use it as surrogate event for kidney survival to investigate the performance of the developed prognostic model with this outcome. We will also assess a potential effect across time (e.g., due to changes in treatment strategies) by including transplantation year as a predictor. Another sensitivity analysis is to assess the prognostic model of the secondary outcome quality of life with quality-of-life data from 24 months after transplantation. Additionally, we can also examine model performance in clinically relevant subgroups. These are paediatric patients, retransplanted patients, multi-organ transplantations, and pre-emptive kidney transplantation [48].

### Model presentation

Reporting will adhere to the “Transparent Reporting of a Multivariable Prediction Model for Individual Prognosis or Diagnosis” (TRIPOD) recommendations [49]. In particular, the final models for the primary and secondary outcomes, respectively, will be presented using the coefficient estimates and 95% confidence intervals, and will include the covariance matrix of the random effects, the error variance, and the regression formula to allow independent application and validation of the model.

## Discussion

We described the study protocol for the planned development and validation of a novel clinical prediction model for kidney graft survival (primary outcome) and quality of life and eGFR slope (secondary outcomes) in the Swiss transplant setting. Our methodological approach will be based on a multivariable Fine and Gray model and two linear mixed models for the primary and secondary outcomes, respectively. Our proposed statistical procedures will take into consideration dependencies in the data (same-donor kidney transplants), between-center heterogeneity, and competing risks.

Previous studies on prognostic models and risk scores related to kidney transplantation outcomes cannot easily be applied into clinical practice in Switzerland; thus, no risk score is currently routinely applied in the deceased-donor organ offer. Among the main reasons are differences in the patient population and transplant setting: for example, in Switzerland hepatitis C status and ethnicity, two widely used prognostic factors (e.g., in the OPTN’s KDPI calculator) have too little variability as a prognostic factor in the Swiss population of deceased donors. Also, more marginal donor kidneys are being transplanted compared to the USA. Thus, existing prognostic models and related risk scores need to be carefully assessed and validated before implemented in clinical practice.

The high-quality data collected in Switzerland during organ allocation (demographic data, medical history, immunological data, etc.) and the nationwide multicenter prospective cohort study enrolling all transplant recipients in Switzerland since 2008 is the most ideal setting to develop a novel prediction model. In this process, we will use expert knowledge (four transplant nephrologists and two kidney recipients) to preselect the candidate predictors. Purely data driven methods such as stepwise selection may render too optimistic and therefor underperform with new data [43].

In shared clinical decision-making, adequate communication of the risks tailored to the specific patient is essential. This research project actively involves kidney transplant recipients in the design, the variable selection, and the applicability and interpretation of the novel risk prediction tool.

The potential risk of selection bias in our study is mitigated using a national multi-center cohort that enrolled all transplant recipients in Switzerland and has informed consent of 93% of the population of solid-organ transplanted recipients [6]. The potential risk of model optimism and overfitting is addressed by our sample size calculation that determined the number of parameters that are feasible to fit in our modeling, bootstrap resampling, and by internal-external validation procedure. The approach will enable us to study between center heterogeneity if present.

The long-term goal of this research proposal is to deliver a risk calculator as a tool that is applicable in clinical practice to assist clinicians and their patients in their informed decision-making. In the future, healthcare providers together with patients, for example, can predetermine the risk they are willing to accept from a donor kidney, with graft survival, quality of life, and eGFR slope estimates available for their consideration. Approval from health regulatory authorities need to be considered as well. Furthermore, subsequent studies can perform external validation with data from other countries and assess the clinical impact of the novel prognostic tool.

## Conclusion

The prediction model for the prognosis of kidney graft survival, quality of life (patient-reported overall health), and eGFR slope will use data from a national multi-center cohort study and the Swiss organ allocation system. By adhering to recently developed best practices in model development, validation, and reporting, we will minimize potential risk of bias and provide a reliable risk assessment in deceased-donor kidney transplantation for nephrologists and their patients.

## Data Availability

Materials (expert survey, list and ranking of selected candidate predictors, and sample size calculation) are available on the Open Science Framework (osf.io/35apn).
